# Development of a defined compost system for the study of plant-microbe interactions

**DOI:** 10.1038/s41598-020-64249-0

**Published:** 2020-05-05

**Authors:** E. Masters-Clark, E. Shone, M. Paradelo, P. R. Hirsch, I. M. Clark, W. Otten, F. Brennan, T. H. Mauchline

**Affiliations:** 1 Sustainable Agriculture Sciences, Rothamsted Research, Harpenden, UK; 2 School of Water, Earth and Environment, Cranfield University, Bedford, UK; 3 Teagasc, Environmental Research Centre, Johnstown Castle, Wexford, Ireland

**Keywords:** Applied microbiology, Soil microbiology

## Abstract

Plant growth promoting rhizobacteria can improve plant health by providing enhanced nutrition, disease suppression and abiotic stress resistance, and have potential to contribute to sustainable agriculture. We have developed a sphagnum peat-based compost platform for investigating plant-microbe interactions. The chemical, physical and biological status of the system can be manipulated to understand the relative importance of these factors for plant health, demonstrated using three case studies: 1. Nutrient depleted compost retained its structure, but plants grown in this medium were severely stunted in growth due to removal of essential soluble nutrients - particularly, nitrogen, phosphorus and potassium. Compost nutrient status was replenished with the addition of selected soluble nutrients, validated by plant biomass; 2. When comparing milled and unmilled compost, we found nutrient status to be more important than matrix structure for plant growth; 3. In compost deficient in soluble P, supplemented with an insoluble inorganic form of P (Ca_3_(PO_4_)_2_), application of a phosphate solubilising *Pseudomonas* strain to plant roots provides a significant growth boost when compared with a *Pseudomonas* strain incapable of solubilising Ca_3_(PO_4_)_2_. Our findings show that the compost system can be manipulated to impose biotic and abiotic stresses for testing how microbial inoculants influence plant growth.

## Introduction

Novel and optimised alternatives to artificial fertilisers, which can be deleterious to the environment, are required to achieve sustainable intensification of agriculture^1^. Plant growth promoting microbes found at the root-soil interface – part of the root microbiome – have an underexploited arsenal of capabilities that can improve plant health, growth and nutrient status^2^. The network of interactions between microbial partners and the host plant is complex and not well understood. This knowledge is essential for the production of plant growth promoting bioinoculants, which are predicted to be an important part of the solution to feeding the world’s growing population^3–5^.

Plant growth promoting rhizobacteria (PGPR) have been shown to increase acquisition of many nutrients and improve plant health and productivity where nutrients may be limiting. These nutrients include iron^6^, phosphorus^7^ and potassium^8^. The ability of PGPR to mitigate abiotic stresses such as drought^9^, salt^10^ or alkaline soil^11^ has been demonstrated in many crop plants. Potato plants exposed to all the aforementioned stresses in combination survived when inoculated with two *Bacillus* species^12^. The mechanism of tolerance, induced by the bacteria, was shown to be an increase in reactive oxygen species scavenging enzymes and an improvement in photosynthetic performance^12^. *Pseudomonas* species are important PGPR, overcoming drought stress in maize^13^ and often possessing many of the characteristic traits of plant growth promotion: production of siderophores, indole-acetic acid and ammonia^14^. Microbiome research is also focusing on the potential of beneficial microbes to improve agricultural resilience and soil health, enhancing practices such as phytoremediation^15^, the creation of disease suppressive soils^16^, and reducing dependency on artificial fertilisers^17^. Bacterial inocula have been shown to reduce incidence of disease across a broad range of both fungal and bacterial diseases, enhanced further when mixed inocula are used^18^.

In order to assess the potential benefits of PGPR there is a need for the development of a defined testing platform. Previous work has used simplified inert substrates, such as sand, glass beads, vermiculite or perlite, but these are less conducive to root growth and are a much less intricate and realistic matrix than soil^7,19^. Conversely, the use of real soil is often unsuitable due to inherent difficulties in altering soil nutrients, without also affecting soil physical and biological status, making the importance of each individual component for plant health difficult to quantify. We argue that sphagnum peat moss compost, hereafter referred to as compost, provides a suitable substrate to test factors that influence plant growth. Other systems are either too simple to replicate *in vivo* conditions well enough to test inoculants, such as vermiculite, or too complex to manipulate nutrient levels, such as soil. Compost is an irregular, organic matter matrix with absorption and adsorption properties that provides  chemical complexity and an excellent structure for plant growth whilst being easy to manipulate and implement stresses. Tomato plants grown in substrates containing peat showed improved growth compared with using other media such as sand and perlite^20^, and growth of daisies was also improved when vermiculite was supplemented with compost and sphagnum moss peat^21^. Compost provides the optimal structure for plant growth, allowing the effect of implemented stresses or microbial inoculants to be measured.

Many experiments use *in vitro* functional screening assays to predict the ability of bacteria to promote plant growth via biotic and abiotic stress resistance. While this is the accepted method, it is important to be able to test that these abilities can be replicated in an *in planta* system^22^. For example, genomic screening of bacteria for plant growth promoting genes is a useful indicator of potential PGPR function. However, plant growth promoting genes are often not expressed *in planta*, perhaps due to an incompatible plant-microbe interaction. It is therefore necessary to test potential inoculants *in vivo* to confirm their plant growth promoting efficacy^23^. In order to develop a system to test microbial interactions *in planta*, it is essential to be able to define the physical matrix and soluble nutrient status of the system, and to understand how changing these factors affect plant growth.

In this work we describe a defined compost system and demonstrate the use of this medium with three case studies: 1) Compost nutrient status – removal and reconstitution; 2) Compost physical structure status – the effect of pore size on plant growth; 3) The deployment of microbial inoculants, with the example of a P solubilising bacterial isolate, to mitigate soluble-P deficiency.

## Materials and methods

Case study 1 tested the impact of washing compost on nutrient status and the potential for this impact to be reversed with the addition of nutrients. The nutrient levels of washed and unwashed compost were obtained, and the growth of several crop plants were tested in this medium. Different methods of reconstituting the nutrient levels of the compost were tested to re-establish wheat biomass to that of the unwashed compost. Case study 2 identified the relative importance of matrix physical structure as well as nutrient status for wheat growth. This involved milling to reduce pore size and growing wheat plants in both washed and unwashed compost. Case study 3 investigated the ability of a P solubilising microbial inoculant to support wheat growth in a soluble P deficient system.

### Removal of soluble nutrients and assessment of washed compost

The compost used was sphagnum peat moss (95%), with added silver sand (5%); with a 0-3 mm particle size; pH 5.3-6.0 using lime; additional fertiliser N-144, P-73 and K-239 mg/L; 0.1 kg/m^3^ Micromax micronutrient fertiliser; and a wetting agent as standard (Levington F2 + sand, supplied by ICL, Ipswich, UK). Compost was washed to remove soluble nutrients by flooding one-part compost to eight-parts tap water, mixing and breaking up any aggregates, and draining through a 0.63 µm sieve. This process was repeated three times. The effects of washing the compost on the component macro- and micronutrients were obtained using three methods: combustion analysis to measure total N, (LECO TruMac combustion analyser – Dumas method), nutrient extraction (i.e. NH_4_, NO_3_ and Olsen P) and X-ray fluorescence. The X-ray fluorescence was carried out on a Bruker Tracer 5i portable X-ray fluorescence (pXRF) spectrometer using Bruker’s EasyCal software. Milled and dried samples were loaded into xrf cups lined with Prolene films.

Bulk density was measured as mass per unit volume, calculated using the mass of oven-dry washed compost and its volume. Compost was packed to the same density in a known volume and then weighed. The pH change of the compost before and after washing was measured, using a 1:2.5 sample: water ratio. Measurements were taken using a PerpHecT ROSS Micro Combination pH electrode (Thermofisher).

### Plant germination and selection of seeds

Seeds were sterilised as described by Robinson *et al*^24^., immersed in 70% ethanol for ten minutes, followed by submersion in sodium hypochlorite (1.5%) for one hour. After washing thoroughly, seeds were left overnight in sterile water at 4 °C in the dark, before choosing seeds of a similar size and placing them on damp sterile germination paper (Anchor, Minnesota 55101, USA) in a Petri dish. These were germinated in the dark at room temperature for three days. For planting, seeds were chosen with a coleoptile of length between 3 and 5 mm. Wheat (Cadenza) was used for all experiments except in supplementary figure S4, where different plant species  were tested in washed and unwashed compost. These were: Wheat (Cadenza), Barley (Atlas), Clover (Merula), Oilseed rape – (OSR, Aries).

### Reconstitution of nutrients in washed compost

Two methods were tested to reconstitute the nutrient content of the washed compost, with the aim of controlling nutrient levels. These enabled the testing of the importance of specific nutrients for plant growth. Firstly, plants in pots (9×9×10 cm, containing 150 g air-dried washed or unwashed compost) were watered daily with 50 ml Hoagland’s solution with or without soluble phosphate^25^ (Supplementary table S1). Secondly, a modified version of Letcombe’s solution (Supplementary table S1) was applied. The differences between the amount of N, P and K in unwashed and washed compost was determined and used to calculate the amount of each nutrient to be added to each pot of washed compost. A single concentrated dose of the primary macronutrients, N, P and K (as required) was applied using a pipette enabling even distribution of the solution over the surface of the compost. This was followed with the addition of 300 ml of micronutrient solution, in staggered doses until all the solution was absorbed by the compost without subjecting the plants to waterlogged conditions. Growing plants were then watered as necessary with tap water.

### Preparation of soluble P deficient compost, supplemented with recalcitrant tricalcium phosphate

Washed compost was supplemented with tricalcium phosphate, Ca_3_(PO_4_)_2_, (Sigma) as an insoluble inorganic P source, at a 200:1 ratio^7^. Once germinated, a single plant was transferred to each pot and plants were watered with 50 ml of a modified Hoagland’s solution (without P) daily.

### Milling of compost to reduce particle size

The effect of the compost particle size on wheat growth was tested. Washed and non-washed compost was air dried before milling using a hammer mill with a 0.5 mm mesh. The compost was re-wetted by submersing the bottom of the pots in water from a tray prior to transplanting seedlings to prevent hydrophobicity issues during plant growth. To ensure the amount of compost was comparable, each pot was allocated 150 g compost prior to milling.

### Plant measurements and harvesting

In all plant experiments, wheat was grown until flowering, n = 7 unless stated otherwise in figure legend. Aerial biomass was harvested and weighed after drying at 80 °C for 36 hours.

### ***In vitro*bacterial phosphate solubilisation assay**

Microbes were isolated in August 2017 as described by Mauchline *et al*.^26^ from the rhizosphere of oat, bean and wheat plants grown at Furzefield, Rothamsted Research (51.809094, -0.380412). This involved vortexing roots in sterile water to sample the rhizosphere soil, which was centrifuged to obtain a pellet. A library of bacterial rhizosphere isolates was obtained, and these were screened *in vitro* for the ability to solubilise an example of an insoluble form of phosphate, Ca_3_(PO_4_)_2_. Pikovskaya medium (PVK) uses an inorganic, insoluble form of P (Ca_3_(PO_4_)_2_) and turns clear when the inoculated strains liberate PO_4-_. The medium was prepared as described by Pikovskaya^27^. Isolates from the library were grown for 48 hours in tryptic soy broth (TSB) (Sigma) at 28 °C in a shaker, and 1 µl of each culture was pipetted onto PVK media, with 12 isolates gridded per plate. Sterile TSB served as a negative control. The inoculated plates were incubated at 25 °C for seven days. To confirm that their ability to solubilise Ca_3_(PO_4_)_2_ was persistent, P-solubilising rhizobacterial isolates were subjected to sub-culturing three times on PVK.

The ability of rhizobacterial isolates to access insoluble phosphate was described by the phosphorus solubilisation index (PSI)^28,29^ as: (colony diameter + halo diameter) / colony diameter. In total, of the 578 bacterial isolations, 82 were capable of solubilising Ca_3_(PO_4_)_2_, and 60 were selected for 16S rRNA gene sequencing, and an additional random selection of 32 isolates unable to solubilise Ca_3_(PO_4_)_2_ were also sequenced as a comparison.

### Microbial DNA isolation, PCR and 16S rRNA gene sequencing

To identify the P solubilising and non-solubilising bacteria, isolates were subjected to 16S rRNA gene sequencing. Bacterial isolates were cultured overnight in TSB at 25 °C. Bacterial DNA was released using microLYSIS-Plus (Microzone, United Kingdom). Lysates were diluted 10-fold prior to PCR.

16S rRNA gene PCR was carried out on microbial DNA isolate extracts using the primers 341f(5’CCTACGGGAGGCAGCAG)^30^ and 1389r (5’ACGGGCGGTGTGTACAA)^31^. PCR reactions were carried out using the ThermoPrime 2x ReddyMix PCR Master Mix Kit (Thermo Fisher Scientific). Once prepared, the samples were placed in a thermocycler for PCR and subjected to the following conditions: 95 °C 1 min, followed by 30 cycles of 95 °C 1 min, 60 °C 1 min, 72 °C 1 min and a final extension step of 72 °C for 5 min. The PCR product was purified (Qiagen, Venlo, Netherlands) prior to sequencing.

Sequencing of 16S rRNA gene PCR products was carried out by Eurofins MWG/Operon (Germany) using a PCR product concentration of 10 ng µl^-1^ with the 341f primer. Sequences produced were tested with the BLASTN algorithm using default settings^32^.

### Bacterial culture, quantification, washing, inoculation

The P-solubilising pseudomonad (Psol) isolate with the highest solubilisation index and a non-P-solubilising pseudomonad (nonPsol) were chosen as they were morphologically and phylogenetically similar and were derived from the same host plant (oat). In addition, the 16S rRNA gene sequence identified them to both belong to the *Pseudomonas fluorescens* species complex. Psol recorded the highest solubilisation index (4.37). The average PSI score of all isolates positive for P solubilisation was 2.71 and the minimum score was 0.72. NonPsol did not solubilise Ca_3_(PO_4_)_2_ *in vitro* returning a score of 0.

To inoculate wheat plants, cultures of each isolate were grown in 50% TSB at 28 °C in a shaker overnight. Cultures were measured spectrophotometrically to confirm that they were in the exponential growth phase (OD_600_ 0.6-0.8). When this was the case, the cultures were spun at 353 x g for ten minutes, the supernatant removed, and cells resuspended in Ringer’s solution. Both cultures were diluted using Ringer’s solution to OD_600_ 0.1 to ensure that a consistent 10^8^ cells were applied per pot. For each plant, 1 ml of the Ringer’s diluted culture was applied onto the root, ten days after planting. A control treatment using no bacterial inoculant was included in this experiment.

### Statistical analysis

Significance was defined as *p* < 0.05 using a two-way ANOVA and Tukey HSD post-hoc tests for pairwise comparisons. All statistical calculations were carried out in R and all assumptions of the statistical tests were met, n = 7 unless stated otherwise.

## Results

### Effect of washing compost on soluble N, P and K and other elements

**Figure 1:**
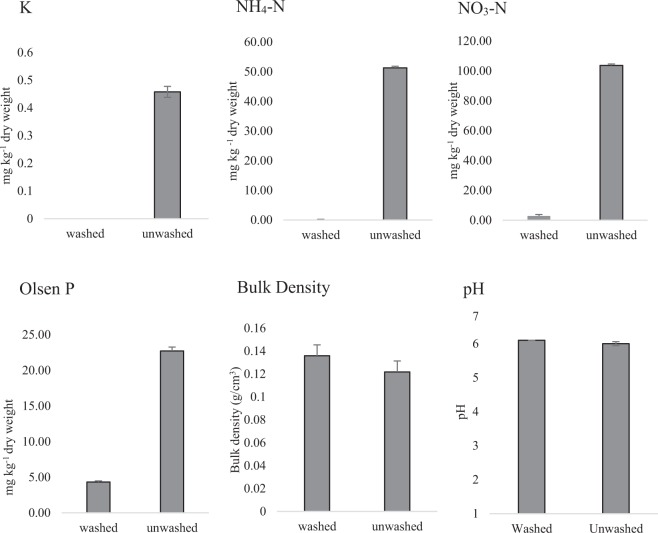
Nutrients, bulk density and pH in washed and unwashed compost. Extracted nutrient concentrations are expressed as mg kg^−1^ dry weight soil. Bulk density was measured as mass per unit volume, calculated using the mass of oven-dry compost (Md) and its volume (V), using the formula Db = Md/V. Bars show standard error.

Nutrient analysis showed that, of the total nutrients, washing compost removes: 100% of K, 99.7% NH_4_, 97.1% of NO_3_ and 80.96% of P (Fig. 1). Other secondary macro- and micronutrients were included in the analysis; washing reduced the levels of, for example, magnesium, aluminium and sulphur (Supplementary table S2).

### Effect of washing compost on bulk density and pH

Washing the compost did not change its bulk density or pH, compared to unwashed compost (Fig. 1). The pH of the compost in many of the different nutrient configurations was tested; all nutrient treatments were not significantly different from the average pH within the given range of the unwashed compost (5.3-6.0) (Supplementary figure S3).

### N, P and micronutrient reconstitution

**Figure 2:**
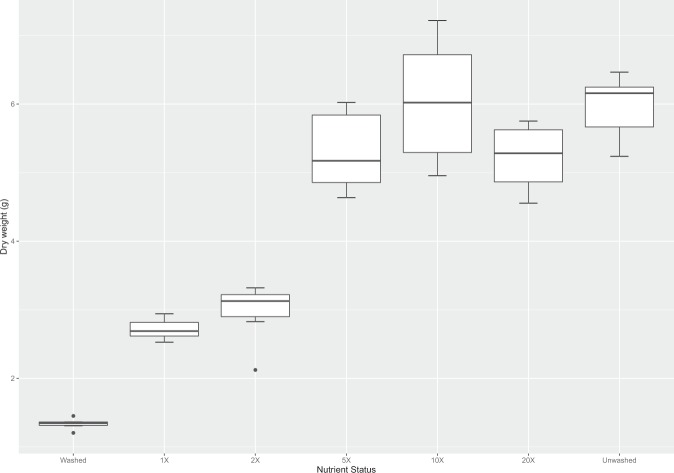
Biomass (as dry weight) of wheat grown in washed compost given nutrients from 1X, 2X, 5X, 10X and 20X the amounts removed by washing. Dry weight is the aerial biomass of wheat harvested at flowering. Central line represents the median, n = 6. Different letters indicate significant difference (*p* < 0.05).

Growth of plants in washed compost was significantly reduced in the four crops (Supplementary figure S4). However, adding the predicted required amount, 1X, of each nutrient as calculated from the nutrient analysis (Supplementary table S2) to replace what had been removed by washing was insufficient to adequately recover plant growth (Fig. 2). We found that it was necessary to supply 5X the amount of soluble N and P to the washed compost (Fig. 2) in order to fully recover plant biomass to the level of plants grown in unwashed compost. Nutrients were also added at 10 and 20X but these did not result in further increases in plant biomass.

### Biomass of plants in milled compost

**Figure 3:**
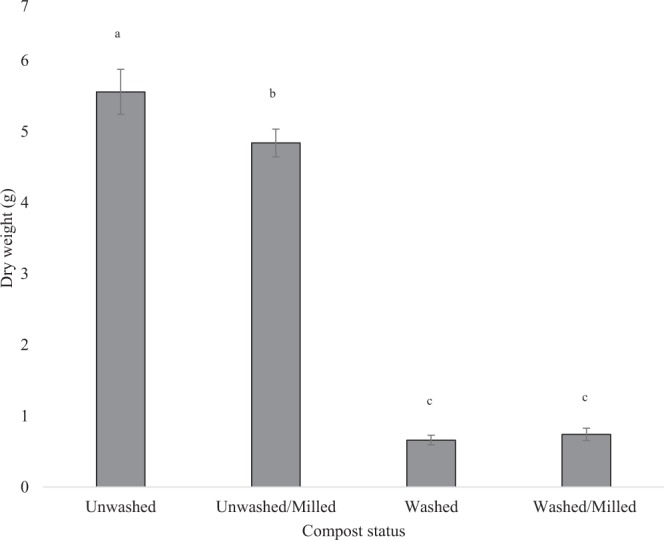
Dry biomass of wheat grown in milled or unmilled, washed or unwashed compost. Plants were grown in the following conditions: Control - unwashed (nutrient replete); Milled - a reduced pore size and replete nutrients; Washed - compost with soluble nutrients removed; Washed/Milled – soluble nutrient deplete and reduced pore size. Wheat above-ground dry biomass was measured at flowering. Bars show standard error, n = 5. Different letters indicate significant difference (*p* < 0.05).

Nutrient status of compost was more important for wheat growth than particle size; reducing particle size reduced growth when nutrients are not limiting. Milling compost reduced the plant biomass in unwashed compost (*p* < 0.05) (Fig. 3). In the case of nutrient depleted compost, the reduction of the particle size did not have a significant effect on the resulting plant biomass (*p* > 0.9). Washing the compost and removing the nutrients had a substantially greater effect on wheat biomass than reducing the particle size.

### Phosphorous solubilising bacteria influence wheat biomass

**Figure 4:**
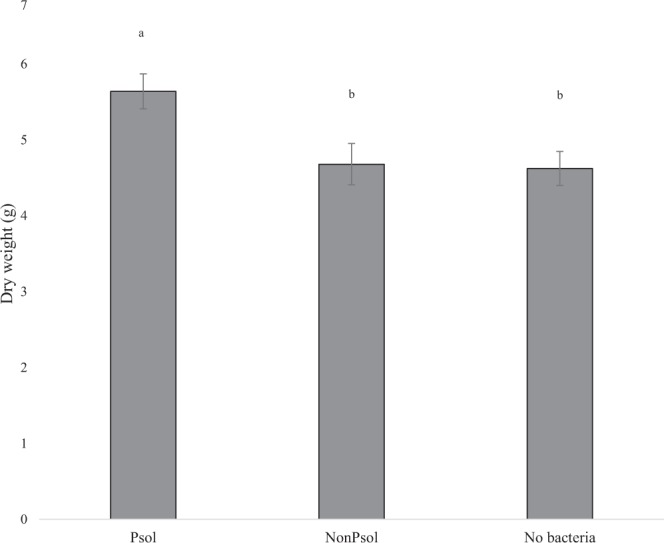
Wheat, with tricalcium phosphate as a source of P, grown with P solubilising or non-P solubilising *Pseudomonas*, or no bacterial inoculant. Psol is a P solubilising *Pseudomonas* species isolate, nonPsol is a non-solubilising *Pseudomonas*. Error bars represent standard error, n = 7. Different letters indicate significant difference (*p* < 0.05).

The 16S rRNA gene sequenced *Pseudomonas* sp. bacteria differed in their ability to access recalcitrant P (Ca_3_(PO_4_)_2_); the P-solubilising bacteria increased wheat growth (Fig. 4). The 16S rRNA gene sequencing data informed the choice for both bacterial isolates chosen for this experiment and identified them as *Pseudomonas* sp. The inoculation of a phosphate solubilising (Psol) bacterial isolate was compared to a non-solubilising bacterial isolate (nonPsol) from the same host, and a control treatment with no bacterial inoculant. The mean above ground biomass of the two bacterial treatments was compared using a two-tailed t-test with equal variance (*p* = 0.02) and plants in the Psol treatment had 18.01% more biomass than plants in the nonPsol treatment. NonPsol showed no difference in biomass compared to the no bacterial control (*p* = 0.8), whereas plants inoculated with Psol had a greater biomass than plants with no bacteria (*p* = 0.01).

## Discussion

### Nutrient limited compost system

As expected, washing the compost reduces soluble nutrient availability and plants subsequently grown in this medium have a significantly reduced biomass. Neither the bulk density or pH of the compost changes significantly after washing so the reduction in above ground growth was attributed to the removal of many of the soluble nutrients, primarily N, P and K (Supplementary table S2).

In order to reconstitute the nutrient levels of the washed compost system, two different nutrient solutions were trialled to find the most efficient and successful recovery of plant growth. It is essential that nutrient levels can be easily manipulated to simulate different nutrient deficiencies for use in experiments with plant growth promoting microbes. Hoagland’s solution provided highly effective and reliable recovery of plant growth when used in the phosphate solubilisation bioassay (Supplementary table S1). This method involved daily watering with a prescribed dose of Hoagland’s solution, allowing nutrients to be replaced gradually. Although Hoagland’s solution can easily omit P, the recipe adds many different sources of N, in combination with other micronutrients. As such, this method is not suitable for mineral N depletion experiments. Removing all sources of N would mean many other essential micronutrients that constitute Hoagland’s solution would be eliminated. The second nutrient solution tested was a modified version of Letcombe’s solution for wheat (Supplementary table S1). One major advantage of this approach is that the nutrients are given as one single dose at the beginning of the experiment and thereafter plants are watered with tap water, significantly reducing the workload.

Reconstituting the compost with the exact amount of N and P calculated to have been removed by washing was insufficient to fully recover plant growth. In order to support plant growth to the same biomass level as that of the unwashed compost, it was necessary to add 5X the original amount of nutrients. It is likely that this can be attributed to the loss of fine particles and their associated nutrients during the washing process. These particles have a high surface area:volume ratio and the mineralisation of organic nutrients to inorganic soluble forms will be reduced in washed compost. Two further concentrations were also tested (10X and 20X), but these had no further effect on plant growth and biomass indicating that the system was replete with nutrients.

The nutrient status of the washed compost is more important than pore size for plant biomass (Fig. 2). Washing compost removes nutrients and results in a pronounced reduction in plant biomass. However, when comparing wheat grown in unwashed compost, milling resulted in a reduced wheat biomass, whereas for washed compost milling had no effect on wheat biomass. It may be the case that under nutrient replete conditions that the reduced biomass seen with milling could be attributed to reduced oxygenation of the soil, water logging and compaction affecting root structure^33^, but this is secondary to the effect of nutrient depletion.

The compost system is a more intricate, ecologically complex matrix than for example, sand or hydroponics. This will mean simulations can be closer to those found in the field – the translation of experiments from compost to field may be more realistic than, for instance, *in vitro* experiments or those using an inert mineral substrate such as vermiculite. Additionally, real soil is not suitable for experiments such as these as it is difficult to manipulate their biotic, abiotic and physical states. The development of soils depleted in particular nutrients would take many years to achieve, whereas the compost system presented here can be readily manipulated for the control of these factors. The main application of this system is in testing microbial interactions and their impact on plant health. The ability of microbes, either as singular inoculants or as synthetic communities, to enhance plant growth can be screened in a standardised, easily manipulated system that can simulate many environmental conditions: nutrient deficiencies, altered physical structure, a reduced microbial pool and abiotic stresses such as drought or flooding.

### Influence of phosphate-solubilising pseudomonads on the aerial biomass of wheat in P-deficient compost

The P solubilising bacterial strain was chosen as it had the highest PSI score. The non-solubilising bacterial strain was chosen as it was not able to solubilise P but was the same species, came from the same host and was morphologically similar to the P solubilising strain. When wheat was inoculated with phosphate-solubilising bacteria (*Pseudomonas* sp.), under depleted soluble P condition but supplemented with recalcitrant P, it had increased above ground biomass relative to wheat inoculated with a non-solubilising *Pseudomonas* species, or a no bacteria control. This suggests that the P solubilising *Pseudomonas* sp. was able to liberate P from inorganic, insoluble Ca_3_(PO_4_)_2_, promoting plant growth. The inclusion of a no bacteria control treatment addresses the potential issue of pH difference between agar and compost systems influencing Ca_3_(PO_4_)_2_ solubilisation. The *in vitro* screen used for P solubilisation is at pH 7, whereas the compost system has a pH ~6. As the compost substrate is slightly more acidic than PVK agar, the recalcitrant source of P could be more soluble in the compost system, making PO_4_ in the Ca_3_(PO_4_)_2_ more readily available to the plant. However, our data shows that plants inoculated with bacteria unable to solubilise P and plants grown with no bacterial inoculant have a similar biomass (*p* = 0.8), and the addition of the P solubilising bacterial inoculant increases wheat biomass relative to the other treatments (*p* = 0.02). This demonstrates that it is the addition of Psol that benefits the growth of wheat, either directly or indirectly, in the compost system.

Isolates screened *in vitro* for their ability to solubilise phosphate may not replicate this *in planta*. The use of PVK media and Ca_3_(PO_4_)_2_ as the recalcitrant source of phosphate is well documented^34^, but soils contain many kinds of metal-phosphate sources and other organic P sources, so screening for other P solubilisation mechanisms in the future should also be considered, to replicate conditions found naturally in soil^35^. Rhizosphere competence, the chemical, biological and physical properties of the growth medium, and microbial competition are all factors that may influence the potential of an inoculant to perform the same functions *in planta* that are observed *in vitro*. Isolates screened using this technique, including *Enterobacter* and *Burkholderia* sp. - both displaying a high phosphate solubilising ability *in vitro* - yielded contrasting results *in planta*, either increasing or having no effect on the biomass of sorghum respectively^36^. Our experiment used compost supplemented with Ca_3_(PO_4_)_2_, thus *in vitro* screening using the same phosphate source was appropriate. For use in natural soil systems, the use of mixed communities with a variety of PGPR functions and phosphate solubilisation abilities is likely to give more success in field situations.

The use of plant growth promoting bioinoculants is an exciting avenue for exploration of alternatives to mineral fertilisers in agriculture. Previously published work focusing on phosphate solubilising microbes has conflicting results. For example, pea plants had increased biomass when inoculated with *Pseudomonas* sp., using Ca_3_(PO_4_)_2_ as the recalcitrant P source^7^, and the growth of walnuts was improved by separate inoculation with *Pseudomonas chlororaphis* and *Pseudomonas fluorescens*, previously screened for phosphate solubilisation using PVK media^37^. The ability to manipulate the washed compost system means high-throughput screenings of plant growth promoters under specific conditions, such as nutrient depletion, pathogen challenge or drought, are possible in a consistent and defined matrix.

## Conclusion

Washing compost significantly reduces soluble nutrient pools, including N, P and K, without affecting its structure or pH. We show that nutrient levels can be manipulated to simulate different nutrient deficiencies and study the potential for microbial inoculants to overcome these deficits. The compost provides a standardised testing system defined in some of its biological, chemical and physical attributes. This defined system creates a platform for detailed and complex plant-microbe interactions to be studied and the simple manipulation of nutritional status provides a reliable screening platform for PGPR. The system is closer to conditions found in the field than the commonly used inert mineral substrates or hydroponics, so we believe that results are more relevant for sustainable agriculture. It has great potential as a platform to provide rigorous and reproducible pre-field testing of commercial microbial inoculants to replace or complement mineral fertilizers.

## Supplementary information

Supplementary information.

## Data Availability

The 16S rRNA sequences used in the current study are available in the NCBI GenBank, accession: MT181117 and MT181116.
