# Supplementary material for: Development of a defined compost system for the study of plant-microbe interactions
Source: Sci Rep. 2020 May 5;10:7521. doi: 10.1038/s41598-020-64249-0 (PMC7200721; doi:10.1038/s41598-020-64249-0)
Supplement: Supplementary file 1 — Supplementary information. [file 41598_2020_64249_MOESM1_ESM.docx]

**Development of a defined sphagnum peat-based compost system for the study of plant-microbe interactions**

**E. Masters-Clark^a,b,c^, E. Shone^a^, M. Paradelo^a^, P. R. Hirsch^a^, I. M. Clark^a^, W. Otten^b^, F. Brennan^c^ & T.H. Mauchline^a*^.**

*^a^ Sustainable Agriculture Sciences, Rothamsted Research, Harpenden, UK*

*^b^ School of Water, Earth and Environment, Cranfield University, Bedford, UK*

*^c^ Teagasc, Environmental Research Centre, Johnstown Castle, Wexford, Ireland*

**Corresponding author*

[*tim.mauchline@rothamsted.ac.uk*](mailto:tim.mauchline@rothamsted.ac.uk)

**Supplementary information**

**Table S1. Modified Letcombe’s solution and primary macronutrient concentrations for nutrient re-addition, B: Hoagland’s solution.**

| **A**: Modified Letcombes solution (N and P deficient) – 300 ml per plant | | |
| --- | --- | --- |
| Stock solution | Amount in 1L | |
| 1M MgSO_4_ | 2 ml | |
| x200 Fe EDTA | 5 ml | |
| 1M CaCl_2_ | 10 ml | |
| 0.5M KSO_4_ | 5 ml | |
|  | | |
| *Add 1ml Micronutrient solution per 1L final solution* | | |
| Micronutrient solution | Amount in 1L | |
| Boric Acid | 2.86 g | |
| Manganese chloride.4.H_2_0 | 1.81 g | |
| Zinc Sulphate.7.H_2_0 | 0.22 g | |
| Copper Sulphate.5.H_2_0 | 0.08 g | |
| Molybdic acid | 0.02 g | |
|  |  | |
| Primary macronutrients (N and P, if required) | Amount in 100 ml | Amount per 150 g dry compost |
| KNO_3_ | 16.2 g | 2 ml |
| KH_2_PO_4_ | 1.2 g | 2 ml |
|  |  |  |
| **B**: Hoagland’s solution (50 ml per plant per day)^1^ | | |
| Macronutrients | Amount in 1 L | ml Stock/L |
| 2M KNO3 | 202 g/L | 2.5 |
| 2M Ca(NO3)2•4H2O | 472 g/L | 2.5 |
| x200 Fe EDTA | 15 g/L | 1.5 |
| 2M MgSO4•7H2O | 493 g/L | 1 |
| 1M KH2PO4 (**if required**) | 136 g/L | 1 |
|  |  |  |
| Micronutrients | Amount in 1L | ml Stock/L |
| H3BO3 | 2.86 g/L | 1 |
| MnCl2•4H2O | 1.81 g/L | 1 |
| ZnSO4•7H2O | 0.22 g/L | 1 |
| CuSO4•5H2O | 0.08 g/L | 1 |
| H2MoO4•H2O or | 0.09 g/L | 1 |
| Na2MoO4•2H2O | 0.12 g/L | 1 |

|  |  |  |  | **A** |  |  |  | **B** |
| --- | --- | --- | --- | --- | --- | --- | --- | --- |
| Nutrient | **Na** | **Mg** | **Al** | **P** | **S** | **Cl** | **K** | **Olsen P** |
|  |  |  |  |  |  |  |  |  |
| Washed | 0 | 0.172 | 0.079 | 0.024 | 0.196 | 0 | 0 | 4.32 |
| Unwashed | 0 | 0.444 | 0.18 | 0.144 | 0.313 | 0 | 0.458 | 22.7 |
| Nutrient | **Ca** | **Ti** | **Cr** | **Mn** | **Fe** | **Co** | **Ni** | **NO_3_** |
| Washed | 1.286 | 0.005 | 0.002 | 0.005 | 0.091 | <0.001 | 0 | 2.81 |
| Unwashed | 1.095 | 0.006 | 0.002 | 0.006 | 0.096 | <0.001 | 0 | 103.66 |
| Nutrient | **Cu** | **Zn** | **As** | **Se** | **Br** | **Rb** | **Sr** | **NH_4_** |
| Washed | 0.001 | 0 | <0.001 | 0 | 0 | 0.002 | 0.003 | 0.14 |
| Unwashed | <0.001 | 0 | <0.001 | 0 | 0 | 0.003 | 0.003 | 51.31 |
| Nutrient | **Mo** | **Cd** | **Sb** | **Cs** | **Ba** | **Hg** | **Pb** |  |
| Washed | 0.003 | <0.001 | 0 | 0 | 0.003 | 0 | <0.001 |  |
| Unwashed | 0.004 | <0.001 | <0.001 | 0 | 0.001 | 0 | <0.001 |  |

**Table S2**. Results of the nutrient chemical analysis of washed and unwashed compost. Two methods were used: **A** – X-ray fluorescence, **B** – nutrient extraction and combustion using LECO. Values give the percentage of each nutrient in the sample (mg/kg^-1^). Compost was analysed from both the washed and unwashed regimes.

**Figure S3. pH of washed compost in different nutrient configurations.** Unwashed compost ranges in pH from 5.3-6 given by the supplier, measured in this experiment at an average of 6. The pH was measured using the Rothamsted Research SOP, modified to accommodate the high water-absorbency of compost at a ratio of 1g:2.5 ml deionised water. All treatments are washed compost except unwashed control. Bars give standard error. NS: Modified Letcombe’s nutrient solution containing all necessary macro and micronutrients with the exception of KNO_3_, KH_2_PO_4_ and Ca_3_(PO_4_)_2_. Where there is no error bar, standard error was 0. pH was measured to the nearest 1dp, n=3.


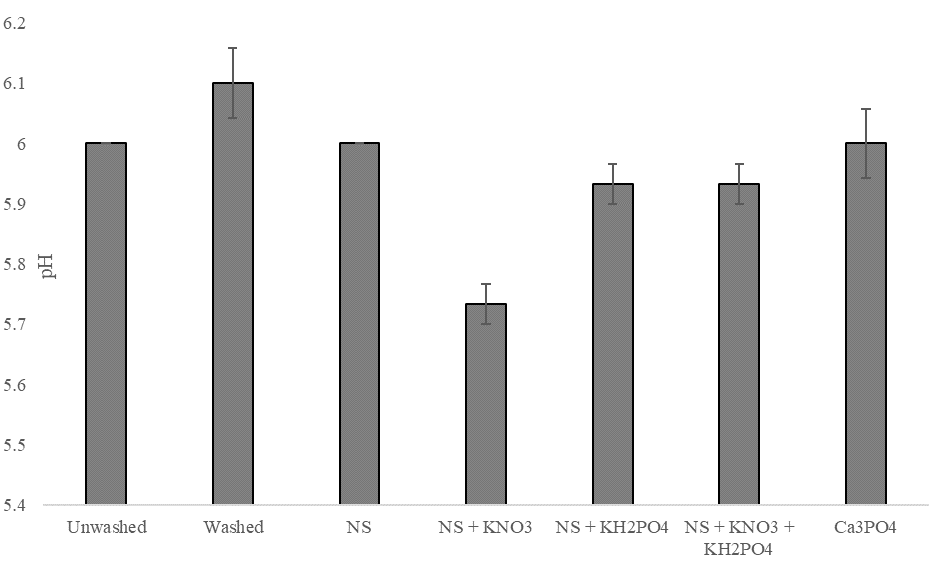


**Figure S4. Biomass of four different crop plants in washed and unwashed compost**. Dry weight (g) biomass of plants grown in different washing regimes. Error bars represent standard error. Plants were dried at 80°C for 36 hours prior to weighing. Barley, Clover, Oilseed rape (OSR) and Wheat were grown for eight weeks before harvest. Biomass of plants grown in washed compost was compared to those grown in unwashed compost using a two-tailed t-test: **B**: *p* <0.01, **OSR**: *p* < 0.01, **W**: *p* <0.02, **C**: *p* <0.04.


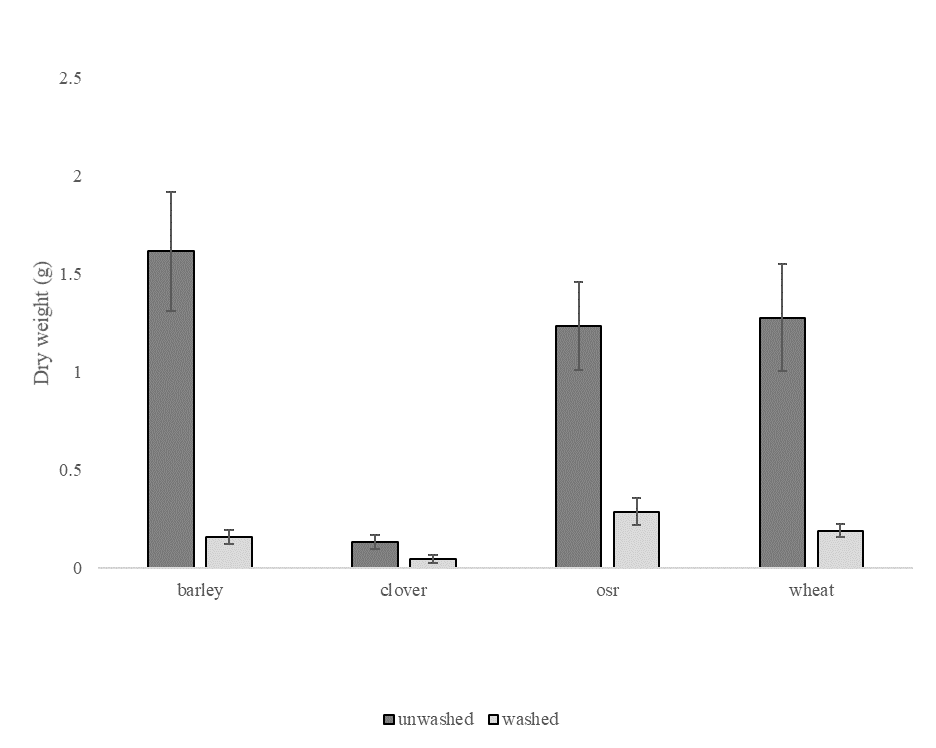


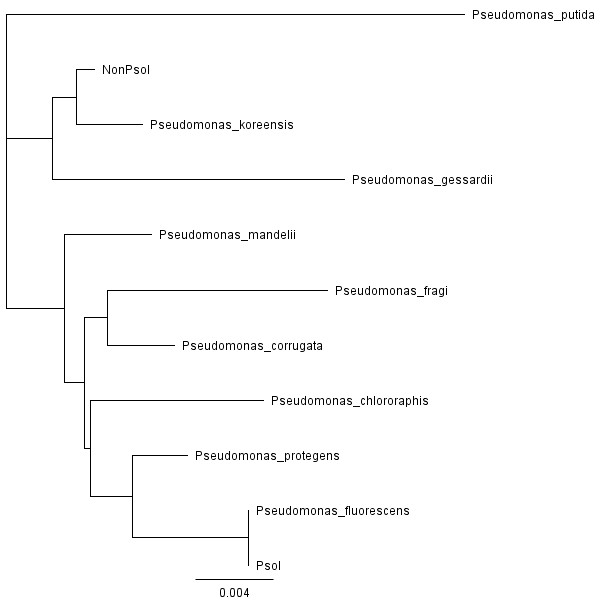


**Figure S5. 16S phylogeny of the *Pseudomonas* isolates used in figure 4.** Affiliation of the sequences of the 16S rRNA gene for the chosen isolates (Psol – the P solubilising isolate, NonPsol – the non-solubilising isolate). Analysis was done using default settings in Geneious (Neighbour-Joining method).

**References**

1. Hoagland, D.R. & D.I. Arnon. The water-culture method for growing plants without soil. *California Agricultural Experiment Station*. **347**(2nd edit) (1950).
